# More Depressive Symptoms, Alcohol and Drug Consumption: Increase in Mental Health Symptoms Among University Students After One Year of the COVID-19 Pandemic

**DOI:** 10.3389/fpsyt.2021.790974

**Published:** 2021-12-16

**Authors:** Ezgi Dogan-Sander, Elisabeth Kohls, Sabrina Baldofski, Christine Rummel-Kluge

**Affiliations:** ^1^Department of Psychiatry and Psychotherapy, University of Leipzig Medical Center, Leipzig, Germany; ^2^Department of Psychiatry and Psychotherapy, Medical Faculty, University of Leipzig, Leipzig, Germany

**Keywords:** COVID-19, university students, mental health, alcohol consumption, depressive symptoms

## Abstract

**Background:** As the majority of studies examining mental health during the pandemic are cross-sectional, little is known about the changes in mental health during the pandemic, especially in university students. Most studies indicate a worsening of mental health conditions. This study aimed to evaluate the mental health status of German university students during the third wave of the pandemic in 2021 and to compare the results to a sample of a congruent cross-sectional study from 2020.

**Methods:** Two cross-sectional and anonymous online surveys among university students were conducted (first survey: July-August 2020, *N* = 3,382; second survey: March-April 2021, *N* = 5,642). Mental health status was assessed with standardized measures (depressive symptoms, alcohol and drug consumption, and eating disorder symptoms), and social and emotional aspects of the COVID-19 pandemic were assessed. In addition to descriptive statistics and group comparisons of the two survey samples from 2020 and 2021, respectively, risk and protective factors related to mental health were analyzed.

**Results:** There were significant differences in severities of depressive symptoms and alcohol and drug consumption between the two online surveys from 2020 and 2021. Findings suggest an increase in the severity of depressive symptoms as well as alcohol and drug consumption. Significantly more respondents reported suicidal ideation in the survey from 2021. Lower self-efficacy, less social support and lower resilience as well as higher perceived stress and more loneliness were reported by the participants of the survey from 2021 compared to 2020. Regarding factors predicting mental health symptoms, being female was a positive predictor for hazardous alcohol use and anorexia nervosa in comparison to men. Further, younger age, being diverse, higher perceived stress and loneliness were positive predictors for all mental health outcomes.

**Conclusion:** This study reveals an increase in severities of depressive symptoms, including suicidal ideation, drug and alcohol consumption among students. Being diverse, younger age, higher perceived stress and loneliness were mutual risk factors for higher depressive and eating disorder symptoms as well as alcohol consumption. Universities and health care policy should recognize and address mental health issues of young adults during ongoing times of crisis and invest in easy-to-access interventions.

## Introduction

The global outbreak of the coronavirus disease 2019 (COVID-19) has brought unprecedented and significant stress to daily life of nearly all people around the globe (1). Governments worldwide mandated several preventive restrictions, also affecting the universities and their students. Students were impacted not just in their academic, but also in their interpersonal occupational functioning.

Previous studies indicated that young adults show a lower risk for serious somatic complications due to COVID-19 (2). However, little is known about the mental health consequences. In comparison to chronic physical disorders, mental disorders have their peak onset mostly during young adulthood (e. g., major depressive episode with a median age-of-onset in the early to mid 20s) (3), which makes university students a vulnerable population. Also, other stressors like academic pressure and increased responsibilities due to separation and individuation from their family may play an important role in vulnerability for mental health problems (4). A recent meta-analysis investigating the prevalence of depressive symptoms among university students in China showed that over one-fourth of students experienced depressive symptoms during the COVID-19 pandemic (5). A cross-sectional survey among university students in France revealed that ~50% of the respondents reported moderate to severe depressive symptoms (6). Several risk factors for depressive symptoms such as loss of employment, older age, more loneliness, higher perceived (study) stress as well as lagging academically behind others were observed in previous cross-sectional studies (7–10).

The prevalence of suicide, a leading cause of death among young adults in high-income countries (11) remained overall relatively unchanged during the pandemic (12–14). However, the literature reporting suicide rates in university students is scarce. O'Connor and colleagues demonstrated that younger adults (18–29 years) reported higher levels of suicidal ideation than older adults during the pandemic in the UK (15). Also, a Centers for Disease Control and Prevention (CDC) survey showed similar results, with young adults having higher rates of suicidal ideation than the general population during the pandemic (16).

Heterogeneous results were reported regarding alcohol consumption among students during the COVID-19 pandemic: While some studies demonstrated an increased frequency of drinking (17, 18), others indicated a decreased frequency (19). On the other hand, a both decreased and increased quantity in alcohol consumption among university students was reported (20, 21). A survey among Israeli and Russian students showed that substance use (tobacco, alcohol, and cannabis) was increased during the pandemic (22). On the contrary, Busse et al. observed unchanged cannabis use during the pandemic (23). It has also been shown that substances were used by some students as a coping mechanism with stress related to the pandemic (24).

The literature regarding change in eating disorder symptoms among university students during the COVID-19 pandemic is scarce. Zhou and Wade (25) showed a significant increase in eating disorder symptoms during the COVID-19 pandemic among female university students. A strong association between problematic eating behaviors and lockdown related stress was also demonstrated by another study among university students (26).

A recent cross-sectional study among college students highlighted that the majority of the participants (61%) reported that the management of preexisting mental health problems was disrupted (27). It is of note that pre-existing psychiatric disorders have been found to be associated with greater increases in stress as well as anxiety during COVID-19 mandatory lockdown (28).

Overall, the majority of the studies examining mental health in university students during the pandemic is cross-sectional in nature. Therefore, information on the magnitude of changes in mental health due to the pandemic as well as causality is scarce. There are some studies comparing the mental health status of university students before and during the pandemic which overall indicate a worsening of mental health conditions. For instance, the prevalence of university students experiencing moderate to severe depression and anxiety increased after the onset of the pandemic (29). In addition, an increase in psychological stress, depression, and anxiety symptoms was demonstrated in another study (30).

One year after the onset of the pandemic many people reported noticing high exhaustion and helplessness, pointed out the uncertainty and not being able to see an end in sight. Nevertheless, there is only little known about the changes in mental health during the pandemic, especially in university students. A comparative analysis of cross-sectional surveys during spring and autumn of 2020, respectively, in university students in Switzerland showed that students in the autumn cohort (October 2020) reported more binge drinking events and less physical activity than those in the spring cohort (April 2020) (31). Furthermore, students in the autumn cohort reported lower self-efficacy and lower social support in comparison to students in the spring cohort, which indicates the negative impacts of a long-lasting pandemic in several dimensions. On the other hand, Reznik et al. demonstrated no significant increase in binge drinking among university students from Russia and Israel surveyed in May and October/November of 2020, respectively (32). In a study from Italy, a significant increase in depressive symptoms during the COVID-19 lockdown was shown in comparison with the results before the lockdown (33). However, a subsequent reduction in depressive and anxiety symptoms was observed after the lockdown.

During the first wave of the pandemic (March–April 2020), German government had mandated several preventive measures, one of them being a lockdown. During the first lockdown period, we conducted a cross-sectional study in July and August 2020 which showed that more than one third of the students reported clinically relevant depressive symptoms; higher perceived stress, lower social support and lack of indirect social contact as well as not being a parent were significant predictors for increased depressive symptoms (10).

After the first wave of the pandemic, a debate about a possible second wave was started by July 2020. First, a partial lockdown was initiated in November 2020. Due to the high infection rates, harder measures were imposed in mid-December, which were extended due to the third wave of the pandemic. In the beginning of May 2021 most of the restrictions were alleviated. The state of Saxony was one of the most affected states in Germany with the most cases per 100.000 inhabitants (5.634/100.000 on April 8, 2021) (34). Given these circumstances, a second cross-sectional study among the students of University Leipzig was planned. The University of Leipzig with ~30,000 students and 14 faculties, ranging from medicine to theology, can be considered representative for a full-scale University in Germany and Europe (10).

The aims of this follow-up study were: (a) to estimate the severity of depressive and eating disorder symptoms as well as alcohol and drug consumption during the third wave of the pandemic in German university students; (b) to compare the severities of the aforementioned mental health symptoms with the severities in the sample of a congruent cross-sectional study from 2020 (10); and (c) to investigate risk and protective factors related to mental health. Based on existing literature we hypothesized that the severity of depressive symptoms, eating disorder symptoms, and alcohol and drug consumption would be increased in comparison to a sample of university students assessed in 2020. It was further hypothesized that a decrease in income, previous mental illnesses, female gender, and social isolation would increase the severity of these mental health issues.

## Methods

### Participants and Procedure

We conducted our first cross-sectional study in July-August 2020 to investigate the mental health status of students during the first lockdown. Overall, *N* = 3,382 students of University Leipzig, Germany, completed the online survey. University Leipzig comprises 14 faculties ranging from medicine to law and it is with almost 30,000 students Saxony's second-largest university. The present cross-sectional study was conducted online between March 16, 2021 and April 26, 2021, during the last weeks of semester break and the first weeks of summer semester in Germany. The same recruitment procedure was applied as in the first study (10): Current enrollment as a university student was the only inclusion criterion, and no exclusion criteria were applied. The recruitment strategy consisted of contact via email and social media channels of the University Leipzig. The Ethics Committee of the Medical Faculty of the University of Leipzig waived approval for this study because of anonymity of the survey (08.03.2021). All participants provided informed consent to participate at the beginning of the anonymous survey.

A total number of *N* = 6,630 university students started the online survey, and *n* = 5,659 participants completed the survey until Patient Health Questionnaire (PHQ-9), covering the main study outcome measures. Of the remaining participants, *n* = 16 indicated not filling in the questionnaire seriously in a validation question. Data of these participants were thus excluded from the analyses. Unplausible data (*n* = 1) was also excluded after manual validation check by descriptives. The final sample comprised *n* = 5,642 participants.

### Measures

The online survey in the present cross-sectional study consisted of slightly changed questionnaires of the first online survey conducted in 2020 (10). Identical with the first survey, the online survey included questions about sociodemographic information (gender, age, family status, being a parent, having pets, migration status), their faculty, socioeconomic information (income [current and before the pandemic] and change in income, residential status), social media use, personal and indirect social contact, and media consumption.

For the statistical analyses, four main groups of studies were created: Faculty of humanities (study of law, philology studies, theology studies, history, arts, and oriental studies), faculty of medicine (study of human medicine and veterinary medicine), faculty of natural science (chemistry and mineralogy studies, mathematics and computer science studies, physics and earth science studies) and faculty of social studies (education studies, life science studies, social science and philosophy studies, sport science studies, economic and management science studies). The students who reported being enrolled in more than one faculty (*n* = 1,045, 18.5%) were distributed to these groups according to a specific order to avoid overlapping in the analyses: First, students enrolled in medical faculties were defined, afterwards natural science and social studies, and finally, humanities faculties.

Furthermore, respondents were asked about their chronic physical illness status as well as contact to medical personnel during the pandemic. Additionally, the following assessments were conducted:

#### Mental Health Measures

To assess severity of depressive symptoms over the last 14 days the Patient Health Questionnaire-9 (PHQ-9) was used. The PHQ-9 is a widely used and validated assessment instrument of depression severity, comprising 9 items based on depression symptoms (35).

Alcohol consumption was assessed using the Alcohol Use Disorders Identification Test (AUDIT-C) (36). Also, change in drinking behavior during the pandemic was assessed. After rephrasing to “drug or substance use,” the AUDIT items and the item on change in consumption during the pandemic were used to assess drug consumption.

To investigate eating disorder symptoms the 5-item Short Evaluation of Eating Disorder (SEED) was used (37). The items were rated on a 5-point Likert scale. Furthermore, body weight and height as well as changes in body weight during the pandemic and lockdown situation were assessed.

For all mental health measures, higher scores indicate higher levels of psychopathology. Moreover, lifetime psychiatric disorders and treatments of these disorders (past and current) were assessed.

#### Social and Emotional Aspects of the COVID-19 Pandemic

Social support was assessed using the five-item ENRICHED Social Support Inventory (ESSI) (38). Items were answered on a 5-point Likert scale with higher scores indicating higher levels of social support. The reliability of the scale was good, with a Cronbach's α = 0.88 and 0.87 in the 2020 and 2021 samples, respectively.

The UCLA 3-Item Loneliness Scale was administered to assess experienced loneliness (39). Items were rated on a 4-point Likert scale. Higher values indicate more loneliness. The reliability of the scale was acceptable, with a Cronbach's α = 0.78 and 0.74 in the 2020 and 2021 samples, respectively.

The 10-item General Self-Efficacy Scale (GSE) was used to explore the general sense of perceived self-efficacy (40). Items were rated on a 4-point Likert scale with higher values indicating higher self-efficacy. The reliability of the scale was good, with a Cronbach's α = 0.87 and 0.86 in the 2020 and 2021 samples, respectively.

The Brief Resilience Scale (BRS) was used to measure resilience, consisting of six items with a 5-point Likert scale (41). A mean score was calculated, with higher scores indicating higher levels of resilience. The reliability of the scale was good, with a Cronbach's α = 0.82 in both the 2020 and 2021 samples, respectively.

The perception of stress was measured using the 4-item Perceived Stress Scale (PSS-4) (42). Items were answered on a 5-point Likert scale with higher scores indicating more perceived stress. The reliability of the scale was acceptable, with a Cronbach's α = 0.77 and 0.74 in the 2020 and 2021 samples, respectively.

Frequency of personal and indirect contacts was assessed with the items used in the first survey, which were developed by the research team (10). These items include: “How many times per week did you personally meet with people (family members, friends, neighbors, etc.) beside your own household during the second lockdown (as from December 2020)?” and “How many times per week did you have indirect contact, e. g., via phone, with other persons (family members, friends, neighbors, etc.) beside your own household?” These items were rated on a 6-point Likert scale from 1 = “not at all” to 6 = “multiple times a day.”

Moreover, affected aspects of lifestyle (social and cultural activity, healthy eating, dating behavior, and sexual activity) during the second lockdown were assessed with a single item each.

### Statistical Analyses

All analyses were conducted using IBM SPSS Statistics version 25.0. The significance levels were set at α = 0.05 (two-tailed). First, descriptive statistics were performed for sociodemographic and socioeconomic variables as well as mental health outcomes and ratings on social and emotional aspects of the pandemic. Second, one-way analysis of variance (ANOVA) was used to investigate the differences in age, gender, mental health outcomes as well as social and emotional aspects of the pandemic between the first and the second survey. Furthermore, chi-square tests were performed to estimate the differences between categorical variables (suicidal ideation based on item 9 of PHQ-9, changes in drug and alcohol consumption). All effect sizes were interpreted as suggested by Cohen (43).

Because of the anonymity condition, it was not possible to identify the participants who participated in the online survey in 2020. However, as assessed via self-report, 34.8% (*n* = 1,965) stated that they had participated in the first survey. Therefore, all analyses on group differences between the surveys from 2020 and 2021, respectively, were repeated excluding these participants to take the partially paired design into account. Finally, multiple linear regression models were performed to estimate the relationship between mental health outcomes and sociodemographic variables as well as social and emotional aspects. The dependent variables investigated were PHQ-9 sum score, AUDIT-C sum score, and the SEED sum score for anorexia nervosa, respectively. The predictors consisted of age, gender, being a parent, residential and marital status, having a pet, faculties enrolled, migration background, change in financial income, direct and indirect contacts during the pandemic, somatic and psychiatric conditions prior to the pandemic as well as opinion about negative and positive aspects of the pandemic, and scores of the following questionnaires: PSS-4, UCLA, BRS, GSE, and ESSI. For these analyses, categorical variables were converted to dummy variables (with the following reference groups: being male, being enrolled in humanities faculty, having no migration background, having no changes in income, having more than 3 times direct and indirect contact in a week). Outliers were examined and treated where necessary. The assumption of no or little multicollinearity was not violated (Variance Inflation Factor [VIF] >10; correlation matrix check, *r* ≤ 0.85).

## Results

### Participant Characteristics

Table 1 shows the sociodemographic characteristics as well as descriptive results on PHQ-9, UCLA, ESSI, and PSS-4 sum scores of the total sample. As 69.4% (*n* = 3,915) of the participants were female, almost 30% were male (29.1%, *n* = 1,642) and 1.5% (*n* = 85) diverse with an age range of 17–70 years (*M* = 23.47, *SD* = 4.46). In total, 34.8% (*n* = 1,965) stated that they had participated in the first survey from 2020, while 42.6% (*n* = 2,405) of the participants reported not to have participated the first survey and 22.5% (*n* = 1,272) did not know if they had participated in the first survey.

**Table 1:** Characteristics of the total sample (*N* = 5,642).

	**(*n*, %)**	Depressive symptoms ^**^ ***M (SD)***	Perceived stress^*^, ^**^M (SD)	Social support^**^ ***M (SD)***	Loneliness^**^M (SD)	Self-efficacy^**^ ***M (SD)***
Total	5,642	10.1 (5.84)	7.9 (3.08)	20.8 (3.91)	6.21 (2.07)	28.0 (4.50)
**Gender**						
Female	3,915 (69.4)	10.4 (5.81)	8.1 (3.03)	21.2 (3.61)	6.4 (2.01)	27.7 (4.40)
Male	1,642 (29.1)	9.2 (5.75)	7.3 (3.1)	20.0 (4.42)	5.8 (2.14)	28.9 (4.57)
Diverse	85 (1.5)	14.2 (5.92)	9.4 (3.06)	19.4 (4.11)	6.7 (2.21)	26.14 (5.10)
**Age**						
<20	660 (11.7)	10.5 (5.87)	7.9 (3.05)	20.6 (3.86)	6.6 (1.95)	27.8 (4.40)
20–25	3,744 (66.4)	10.1 (5.80)	7.9 (3.06)	21.0 (3.84)	6.3 (2.03)	27.98 (4.42)
26–30	858 (15.2)	10.1 (5.93)	7.8 (3.12)	20.8 (4.01)	5.9 (2.11)	28.1 (4.85)
≥31	380 (6.7)	9.5 (5.96)	7.7 (3.28)	20.1 (4.41)	5.8 (2.33)	28.7 (4.65)
**Marital status**						
In a relationship	2,708 (48 0.0)	9.6 (5.73)	7.6 (3.08)	22.3 (2.88)	6.0 (2.09)	28.2 (4.49)
Single	2,934 (52.0)	10.5 (5.90)	8.2 (3.06)	19.5 (4.22)	6.4 (2.02)	27.8 (4.51)
**Residential status**						
Alone	1,240 (22.0)	10.9 (6.04)	8.2 (3.12)	19.75 (4.43)	6.4 (2.13)	27.8 (4.55)
Not alone	4,402 (78.0)	9.9 (5.76)	7.8 (3.07)	21.1 (3.70)	6.2 (2.05)	28.1 (4.49)
**Being a parent**						
Yes	243 (4.3)	8.2 (5.59)	7.0 (3.14)	21.2 (3.82)	5.7 (2.30)	29.6 (4.26)
No	5,399 (95.7)	10.1 (5.83)	7.9 (3.07)	20.8 (3.92)	6.23 (2.05)	27.96 (4.50)
**Having a pet**						
Yes	970 (17.2)	10.3 (6.13)	8.0 (3.22)	20.9 (4.04)	6.1 (2.20)	27.9 (4.63)
No	4,672 (82.8)	10.1 (5.77)	7.9 (3.05)	20.8 (3.89)	6.24 (2.04)	28.06 (4.47)
**Migration background**						
Self	284 (5.0)	11.3 (6.37)	8.3 (2.93)	19.8 (4.70)	6.1 (2.17)	28.2 (5.20)
Parents	364 (6.5)	11.8 (6.32)	8.4 (2.98)	20.2 (4.03)	6.5 (1.96)	28.1 (4.83)
No migration background	4,994 (88.5)	9.9 (5.74)	7.8 (3.09)	20.9 (3.84)	6.2 (2.07)	28.0 (4.43)
**Income before COVID-19 lockdown**						
No income	1,152 (20.4)	10.2 (5.99)	7.9 (3.15)	20.6 (3.99)	6.4 (2.10)	27.5 (4.57)
1–499 €/mo	1,173 (20.8)	10.4 (5.79)	8.1 (2.99)	20.7 (3.98)	6.4 (2.00)	27.7 (4.29)
500–999 €/mo	2,250 (39.9)	10.1 (5.76)	7.9 (3.07)	20.97 (3.80)	6.2 (2.04)	28.1 (4.50)
≥1,000 €/mo	878 (15.6)	9.6 (5.87)	7.5 (3.12)	21.0 (3.93)	5.9 (2.13)	28.9 (4.44)
No answer	189 (3.3)	9.5 (5.94)	7.7 (3.03)	20.2 (4.19)	6.1 (2.19)	28.3 (5.00)
**Change in income since 03/2020**						
Decrease	957 (17.0)	11.9 (6.02)	8.7 (3.0)	20.2 (4.30)	6.6 (1.92)	27.8 (4.64)
No change	4,302 (76.2)	9.8 (5.72)	7.8 (3.06)	20.9 (3.82)	6.2 (2.07)	28.0 (4.44)
Increase	383 (6.8)	9.1 (5.82)	7.1 (3.07)	21.3 (3.76)	5.6 (2.25)	28.9 (4.71)
**Current income**						
No income	1,241 (22.0)	10.5 (5.87)	8.1 (3.16)	20.5 (4.05)	6.4 (2.01)	27.5 (4.52)
1–499 €/mo	1,143 (20.3)	10.7 (5.95)	8.3 (3.01)	20.6 (3.99)	6.4 (2.03)	27.5 (4.41)
500–999 €/mo	2,273 (40.3)	10.7 (5.95)	7.9 (3.01)	21.0 (3.76)	6.2 (2.02)	28.1 (4.42)
≥1,000 €/mo	824 (14.6)	8.8 (5.49)	7.1 (3.14)	21.3 (3.86)	5.8 (2.19)	29.3 (4.43)
No answer	161 (2.9)	9.4 (6.10)	7.5 (3.02)	20.2 (4.35)	6.0 (2.24)	28.4 (5.02)

* *Reduced sample size of n = 5,588 participants due to missing data*.

** *Depressive symptoms were assessed by PHQ-9 (Patient Health Questionnaire 9), perceived stress by PSS-4 (4-item Perceived Stress Scale), social support by ESSI (ENRICHED Social Support Inventory), loneliness by UCLA (3-Item Loneliness Scale), and self-efficacy by GSE (General Self-Efficacy Scale)*.

Most of the students (43.1%, *n* = 2,391) were currently being enrolled in social studies. As 12.5 % (*n* = 695) reported being enrolled in the faculty of medicine, 16.6% (*n* = 922) stated being enrolled in the faculty of natural science and 27.7% (*n* = 1,539) in the faculty of humanities.

In total, 15.9% (*n* = 886) of the respondents indicated having a chronic medical condition. Only 4.1% (*n* = 231) reported to not being able to keep appointments with their general practitioner or a medical specialist in the last few weeks due to corona restrictions, whereas 48.8% (*n* = 2,756) were able to keep appointments unchanged. 19.8% of the participants (*n* = 1,105) indicated having a history of a mental disorder, of those, 52.7% (*n* = 582) reported having a diagnosed unipolar depression and 20.5% (*n* = 226) an eating disorder (answer format allowed indicating more than one disorder). In total, only 42.3% of participants with a history of a mental disorder (*n* = 467) reported currently receiving treatment (medication and/or psychotherapy).

### Social and Emotional Aspects of the COVID-19 Pandemic

During the period of the second contact ban (from December 2020) most of the participants (62.5%, *n* = 3,529) reported to have had direct personal contact with people outside their household on one or two days per week. Only 17.4% (*n* = 981) indicated not having any direct contact, and 20.1% (*n* = 1,132) stated having direct contacts more than three days a week. Regarding the indirect contacts (e. g., via telephone) with people outside of the own household only 1.4% (*n* = 80) of the respondents indicated not having any indirect contact at all. Another 22.8% (*n* = 1,285) reported having indirect contacts one or two days a week, while the majority of participants (75.9%, *n* = 4,277) stated having indirect contacts more than three days a week.

Regarding lifestyle changes due to restrictions the majority of respondents indicated feeling restricted in the following areas: physical activity (79.1%, *n* = 4,399), cultural activity (96.6%, *n* = 5,372), social activity (98.2%, *n* = 5,459), and hobbies (90.4%, *n* = 5,024). On the other hand, most participants reported not to have been affected by the restrictions in the following areas (or these areas were not applicable to them): sexual activity (51.0%, *n* = 2,834), dating behavior (51.4%, *n* = 2,854), alcohol and drug consumption (65.6%, *n* = 3,645), and healthy eating (61.0%, *n* = 3,389).

Overall, participants scored significantly less in GSE, ESSI, and BRS compared to the first survey in 2020 (see Table 2), indicating a significantly lower self-efficacy, less social support, and lower resilience than participants from the first study (all *p* < 0.001). Further, participants in this study (2021) scored significantly higher in PSS-4 and UCLA (all *p* < 0.001), indicating significantly higher perceived stress levels and more loneliness than in the first survey in 2020.

**Table 2:** Mean scores of instruments measuring social and emotional aspects and mean differences between the two surveys.

	**Survey 2020 *M* (*SD*)**	**Survey 2021** ***M* (*SD*)**	**Result**	***p***	**partial η^2^**
BRS	3.16 (0.76)	3.10 (0.76)	*F*_(1, 8965)_ = 13.92	< 0.001	0.00
ESSI	21.40 (3.70)	20.83 (3.91)	*F*_(1, 9022)_ = 46.97	< 0.001	0.01
GSE	28.57 (4.46)	28.03 (4.50)	*F*_(1, 9022)_ = 31.02	< 0.001	0.00
PSS-4	7.35 (3.17)	7.88 (3.08)	*F*_(1, 8965)_ = 61.70	< 0.001	0.01
UCLA	3.98 (2.20)	6.21 (2.07)	*F*_(1, 9022)_ = 6060.48	< 0.001	0.07

*BRS, Brief Resilience Scale; ESSI, ENRICHED Social Support Inventory; GSE, General Self-Efficacy Scale; PSS-4, Perceived Stress Scale; UCLA, Three-Item Loneliness Scale*.

### Mental Health Outcomes and Differences Between Surveys

The mean PHQ sum score was 10.09 (*SD* = 5.84). In total, 83.4% (*n* = 4,706) of the respondents reported not having suicidal thoughts or thoughts of hurting themselves. On the other hand, 16.5% (*n* = 936) of the respondents stated having these thoughts at least several days in the last two weeks. The ANOVA showed significant differences in PHQ-9 scores between the two surveys, *F*_(1,9022)_ = 133.22, *p* < 0.001, partial η^2^ = 0.02, indicating that participants reported significantly more depressive symptoms in the second survey (2021) than the students participating the first survey in 2020. Significantly more respondents reported suicidal ideation in the second than in the first survey, χ^2^(1, 9024) = 7.02, *p* = 0.008, ϕ = 0.03, small effect.

The mean value of the AUDIT-C sum score was 2.76 (*SD* = 2.19). Participants from the first study in 2020 scored significantly lower in the AUDIT-C in comparison to the participants from the second study, *F*_(1,8968)_ = 4.91, *p* = 0.027, partial η^2^ = 0.00. While 19.3% (*n* = 1,077) of participants reported not consuming any alcohol at all, 80.7% (*n* = 4,511) of respondents indicated drinking alcohol. Of those, 45.9% (*n* = 2,071) indicated consuming more during the pandemic than before, while only 18.0% (*n* = 811) reported consuming less. The chi square test was statistically significant, with the participants from the second study reporting consuming more alcohol during the pandemic than the participants from the first study, χ^2^(2, 7280) = 403.43, *p* < 0.001, ϕ = 0.24, while significantly more respondents from the first study indicated consuming less alcohol than respondents from the second study.

A substantial amount of the students reported not consuming drugs (*n* = 4,559, 81.8%), while only 18.2% of respondents stated currently consuming drugs (*n* = 1,019). Participants from the second study reported consuming significantly more drugs (*M* = 1.34, *SD* = 0.86) than the participants from the first study, *M* = 1.30, *SD* = 0.79; *t*_(8967)_ = −2.38, *p* = 0.017. Regarding the changes in drug consumption during the pandemic significantly more students reported to consume less in the second study in comparison to the first study, χ^2^(2, 1582) = 10.43, *p* = 0.005, ϕ = 0.08, while there was no significant difference between the number of students consuming more drugs (*p* > 0.05).

The mean score of the SEED regarding anorexia nervosa was 0.53 (*SD* = 0.35). The mean score of the SEED regarding bulimia nervosa was 0.36 (*SD* = 0.44). The SEED scores regarding bulimia nervosa did not differ significantly between studies, *F*_(1,8966)_ = 0.32, *p* = 0.574, partial η^2^ = 0.00. A comparison of SEED scores regarding anorexia nervosa was not possible, because information on body weight and height had not been gathered during the first study so that a SEED score could not be calculated.

The two samples did not differ in gender, χ^2^(2, 9024) = 1.70, *p* = 0.428, however, there were significant age differences, *F*_(1,9021)_ = 27.73, *p* < 0.001 (survey 2020: *M* = 23.98, *SD* = 4.64; survey 2021: *M* = 23.47, *SD* = 4.46). Therefore, age was included as a covariate in all analyses on group differences (PHQ-9, AUDIT-C, SEED bulimia nervosa, BRS, ESSI, GSE, PSS-4, UCLA) using analysis of covariance (ANCOVA). The results did not differ when taking age into account.

When excluding *n* = 1,965 participants who stated to have participated in the previous survey, the results on group comparisons did not differ, with the exception of AUDIT-C scores, which yielded no significant differences between the surveys from 2020 and 2021, respectively, *F*_(1,7015)_ = 0.80, *p* = 0.371.

### Regression Models for the Mental Health Outcomes

To examine predictors of mental health outcomes (depressive symptomatology, alcohol consumption, and anorexia nervosa symptoms, respectively) multiple regression analyses were performed.

Higher levels of depressive symptoms were significantly predicted by younger age (*p* < 0.001), being diverse in comparison to being male (*p* = 0.001), living alone (*p* = 0.001), being single (*p* < 0.001), not being enrolled in medicine (*p* < 0.001), natural science (*p* < 0.001) or social studies faculties (p < 0.001) in comparison to being enrolled in humanities faculties, having a migration background (self, *p* = 0.002, or through parents, *p* < 0.001), having a decreased income (*p* < 0.001), having no indirect contacts (*p* = 0.034), not finding positive aspects of the pandemic (*p* < 0.001), having chronic somatic conditions (*p* = 0.011) and a psychiatric disorder (*p* < 0.001) prior to the pandemic, higher perceived stress (*p* < 0.001) and loneliness (*p* < 0.001) as well as lower resilience (*p* < 0.001), self-efficacy (*p* < 0.001) and social support (*p* < 0.001, see Table 3). The results of the regression indicated that the predictors explained 56.2% of the variance, *R*^2^ = 0.56, *F*_(27,5539)_ = 263.32, *p* < 0.001. No statistically significant associations were detected between depressive symptomatology and being a parent, being female in comparison to being male, having a pet, having no or few direct contacts in comparison to having direct contacts more than 3 times a week, having 1–2 indirect contacts a week in comparison to having more than 3 indirect contacts, and finally, finding negative aspects of the pandemic (all *p* > 0.05).

**Table 3:** Linear regression analysis for predictors of depressive symptomatology measured by PHQ-9 (Patient Health Questionnaire-9).

**Variable**	**Unstandardized B**	**Standardized Beta**	**95% CI**	***T***	***p***
Age	−0.07	−0.06	12.11, 16.89	−5.25	< 0.001
**Gender**					
Female	−0.03	0.00	−0.27, 0.21	−0.22	0.828
Diverse	1.44	0.03	0.58, 2.30	3.27	0.001
Being a parent	0.00	0.00	−0.58, 0.58	0.00	0.999
Residential status	−0.42	−0.03	−0.68, −0.17	−3.28	0.001
Having a pet	−0.08	−0.01	−0.35, 0.19	−0.57	0.566
Marital status	−0.50	−0.04	−0.73, −0.28	−4.33	< 0.001
**Faculties enrolled**					
Medicine	−0.81	−0.05	−1.16, −0.48	−4.50	< 0.001
Natural Science	−0.66	−0.04	−0.98, −0.34	−4.04	< 0.001
Social Studies	−0.25	−0.02	−0.50, 0.00	−1.99	0.046
**Migration background**					
Self	0.76	0.03	0.28, 1.23	0.03	0.002
Parents	0.79	0.03	0.37, 1.20	0.03	< 0.001
**Change in income**					
Decreased	0.72	0.05	0.44, 1.00	0.05	< 0.001
Increased	0.27	0.01	-0.14, 0.67	0.01	0.202
**Direct contacts**					
No contacts	0.10	0.01	−0.25, 0.44	0.55	0.580
1–2 times/week	0.12	0.01	−0.14, 0.38	0.88	0.377
**Indirect contacts**					
No contacts	0.93	0.02	0.07, 1.79	2.12	0.034
1–2 times/week	0.04	0.00	−0.21, 0.28	0.29	0.771
Chronic somatic conditions	−0.36	−0.02	−0.65, -0.08	−2.53	0.011
Psychiatric conditions prior to pandemic	−1.24	−0.09	−1.51, −0.97	−8.90	< 0.001
Positive aspects of the pandemic	0.65	0.05	0.42, 0.87	5.71	< 0.001
Negative aspects of the pandemic	−0.16	−0.01	-0.70, 0.38	−0.59	0.554
Resilience	−0.80	−0.11	−0.97, −0.63	−9.17	< 0.001
Self-Efficacy	−0.09	−0.07	−0.12, −0.07	−6.23	< 0.001
Social Support	−0.16	−0.10	−0.19, −0.12	−9.59	< 0.001
Loneliness	0.45	0.16	0.39, 0.51	14.61	< 0.001
Perceived Stress	0.84	0.44	0.79, 0.88	37.19	< 0.001

*Regression sample size n = 5,567*.

The positive predictors for higher levels of harmful alcohol use were younger age (*p* = 0.004), not being female (*p* < 0.001) or diverse (*p* = 0.001), not being a parent (*p* < 0.001), living alone (*p* < 0.001), not having a pet (*p* = 0.001), not being enrolled in natural science faculties (*p* < 0.001) in comparison to being enrolled in humanities faculties, not having a chronic somatic condition (*p* = 0.009), finding both positive (*p* < 0.001) and negative (*p* < 0.001) aspects in the pandemic, having more direct contacts (*p* < 0.001) and finally, higher loneliness (*p* < 0.001), perceived stress (*p* < 0.001), resilience (*p* = 0.004), self-efficacy (*p* = 0.030), and social support (*p* = 0.017, see Table 4). The results of the regression indicated that the predictors explained 16.1% of the variance, *R*^2^ = 0.16, *F*_(27,5521)_ = 33.99, *p* < 0.001. Marital status, being enrolled in medicine or social studies faculties, having a migration background, change in income, frequency of indirect contacts, and having psychiatric conditions prior to the pandemic showed no significant relations to harmful alcohol use (all *p* > 0.05).

**Table 4:** Linear regression analysis for predictors of harmful alcohol use measured by AUDIT-C (Alcohol Use Disorders Identification Test).

**Variable**	**Unstandardized B**	**Standardized Beta**	**95% CI**	***T***	***p***
Age	0.02	0.04	0.01, 0.04	2.91	0.004
**Gender**					
Female	−0.80	−0.17	−0.92, −0.67	−12.68	< 0.001
Diverse	−0.78	−0.05	−1.22, −0.34	−3.47	0.001
Being a parent	1.10	0.11	0.81, 1.40	7.32	< 0.001
Residential status	0.38	0.07	0.25, 0.51	5.75	< 0.001
Having a pet	0.23	0.04	0.09, 0.37	3.23	0.001
Marital status	0.01	0.00	−0.11, 0.12	0.10	0.919
**Faculties enrolled**					
Medicine	−0.12	−0.02	−0.31, 0.05	−1.38	0.168
Natural Science	−0.34	−0.06	−0.50, -0.17	−4.02	< 0.001
Social Studies	0.06	0.02	−0.07, 0.19	0.96	0.335
**Migration background**					
Self	−0.23	−0.02	−0.47, 0.02	−1.81	0.071
Parents	0.09	0.01	−0.13, 0.30	0.80	0.426
**Change in income**					
Decreased	0.07	0.01	0.36, −0.08	0.92	0.356
Increased	0.12	0.01	0.26, −0.09	1.13	0.260
**Direct contacts**					
No contacts	−1.80	−0.32	−1.97, −1.62	−20.18	< 0.001
1–2 times/week	−0.78	−0.18	−0.92, −0.65	−11.44	< 0.001
**Indirect contacts**					
No contacts	0.20	0.01	−0.24, 0.64	0.88	0.377
1–2 times/week	−0.08	−0.01	0.05, -0.20	−1.21	0.226
Chronic somatic conditions	0.19	0.03	0.05, 0.34	2.60	0.009
Psychiatric conditions prior to pandemic	−0.03	−0.01	−0.17, 0.11	−0.46	0.645
Positive aspects of the pandemic	0.36	0.08	0.25, 0.48	6.28	< 0.001
Negative aspects of the pandemic	−0.52	−0.05	−0.80, −0.24	−3.68	< 0.001
Resilience	0.13	0.05	0.40, 0.22	2.87	0.004
Self-Efficacy	0.02	0.04	0.00, 0.03	0.04	0.030
Social Support	0.02	0.04	0.00, 0.04	2.38	0.017
Loneliness	0.07	0.07	0.04, 0.10	4.44	< 0.001
Perceived Stress	0.06	0.09	0.04, 0.08	5.09	< 0.001

*Regression sample size n = 5,558*.

Younger age (*p* = 0.001), being female (*p* < 0.001) and diverse (*p* < 0.014), being single (*p* = 0.001), not being enrolled in medical (*p* = 0.01) and natural science faculties (*p* = 0.006), having once or twice direct contact in comparison to having more direct contacts in a week (*p* = 0.035), lower resilience (*p* = 0.033) and self-efficacy (*p* = 0.003), higher loneliness (*p* < 0.001) and perceived stress (*p* < 0.001) were positive predictors for more symptoms of anorexia nervosa (see Table 5). The results of the regression indicated that the predictors explained 14.3% of the variance, *R*^2^ = 0.14, *F*_(27,5439)_ = 38.67, *p* < 0.001. Being a parent, having a pet, living alone, being enrolled in social studies faculties in comparison to being enrolled in humanities faculties, having a migration background, change in income, frequency of indirect contacts, having no direct contacts in comparison with having more than 3 direct contacts in a week, having chronic somatic conditions, finding negative as well as positive aspects of the pandemic, and finally, social support did not significantly predict symptoms of anorexia nervosa (all *p* > 0.05).

**Table 5:** Linear regression analysis for predictors of anorexia nervosa symptoms measured by SEED (Short Evaluation of Eating Disorder).

**Variable**	**Unstandardized B**	**Standardized Beta**	**95% CI**	***T***	***p***
Age	0.00	−0.05	−0.01, 0.00	−3.02	0.003
**Gender**					
Female	0.18	0.27	0.16, 0.19	19.71	< 0.001
Diverse	0.08	0.03	0.02, 0.15	2.47	0.014
Being parent	0.00	0.00	−0.04, 0.04	−0.08	0.939
Residential status	−0.02	−0.02	−0.04, 0.00	−1.90	0.058
Having a pet	−0.02	−0.02	−0.04, 0.00	−1.84	0.066
Marital status	−0.03	−0.05	−0.05, −0.01	−3.37	0.001
**Faculties enrolled**					
Medicine	0.03	0.04	0.01, 0.06	2.57	0.010
Natural Science	−0.03	−0.04	−0.06, -0.01	−2.74	0.006
Social Studies	−0.01	−0.02	−0.03, 0.01	−1.18	0.239
**Migration background**					
Self	0.02	0.02	-0.01, 0.06	1.16	0.143
Parents	0.02	0.02	-0.01, 0.05	1.47	0.136
**Change in income**					
Decreased	0.02	0.02	−0.01, 0.04	1.49	0.136
Increased	−0.01	−0.01	−0.04, 0.02	−0.58	0.560
**Direct contacts**					
No contacts	0.02	0.03	0.00, 0.05	1.77	0.077
1–2 times/week	0.02	0.03	0.00, 0.04	2.11	0.035
**Indirect contacts**					
No contacts	−0.03	−0.01	−0.09, 0.03	−0.90	0.371
1–2 times/week	−0.02	−0.02	−0.03, 0.00	−1.61	0.108
Chronic somatic conditions	0.00	0.00	−0.02, 0.02	−0.15	0.883
Psychiatric conditions prior to pandemic	−0.05	−0.07	−0.07, -0.03	−5.22	< 0.001
Positive aspects of the pandemic	0.01	0.01	−0.01, 0.02	0.77	0.439
Negative aspects of the pandemic	0.02	0.01	−0.02, 0.06	1.08	0.279
Resilience	−0.01	−0.03	−0.03, 0.00	−2.13	0.033
Self-Efficacy	0.00	−0.05	−.01, 0.00	−2.96	0.003
Social Support	0.00	−0.01	0.00, 0.00	−0.59	0.557
Loneliness	0.01	0.07	0.01, 0.01	4.48	< 0.001
Perceived Stress	0.01	0.11	0.01, 0.01	6.66	< 0.001

*Regression sample size n = 5,462*.

## Discussion

The present study examined the differences in depressive symptoms, eating disorder symptoms as well as alcohol and drug consumption between two online surveys from 2020 and 2021 among university students. Further, the predictive factors of mental health symptoms were investigated. Findings suggest an increase in the severity of depressive symptoms as well as alcohol and drug consumption. Apprehensively, significantly more respondents reported suicidal ideation in the survey from 2021. No significant difference in bulimia nervosa symptoms was observed between the two samples. Lower self-efficacy, less social support and lower resilience as well as higher perceived stress and more loneliness were reported by the participants of the survey from 2021 in comparison to the participants from the survey 2020.

Regarding factors predicting mental health symptoms being female was a positive predictive factor for hazardous alcohol use and anorexia nervosa in comparison to being male, while being diverse was a positive predictor for all mental health outcomes, including depressive symptoms. Also, younger age, higher perceived stress and loneliness were positive predictors for all mental health outcomes. Decrease in income was only a positive predictor for higher depressive symptoms unlike than our hypothesis. Furthermore, having a previous mental illness significantly predicted only the eating disorder symptoms and depressive symptoms. Finally, social isolation (no direct contacts during the pandemic) significantly predicted only lower levels of harmful alcohol use.

Overall, there is a lack of studies focusing on the mental health of university students during the COVID-19 pandemic. Regarding longitudinal data, the majority of the existing literature focused on the pre- to in-pandemic changes of mental health symptoms. An increase in depressive symptoms after the outburst of the pandemic was shown in several studies among university students (29, 30), but also in the general population (44, 45). On the other hand, a recent longitudinal study conducted in Germany on the general population showed that depressive and anxiety symptoms decreased over time (from March/April to June 2020) (46). The result may be explained by the time of last assessment, which was ~2 months later than the first assessment and before the second lockdown. However, our second online survey was conducted during the second lockdown, which was with many restrictions stricter than the first one. Further, the study designs showed differences regarding study sample (study sample consisted of the same participants in the first and last assessment in the study of Bendau et al. (46)).

The factors predicting more depressive symptoms, such as a history of mental illnesses, younger age, lower self-efficacy and social support, were mainly in line with previous studies (31, 46, 47). In the first study, we have found that not being a parent as well as having indirect contacts 1–2 times a week, higher perceived stress, lower social support, and lower self-efficacy predicted higher levels of depressive symptoms (10). However, in the present study not being a parent and having indirect contacts did not significantly predict depressive symptoms.

Contrary to our results, Efstathiou et al. (48) reported no significant change in suicidal ideation of respondents between the first and second lockdown period in Greece (48). Further, another study reported a decrease in suicidal ideation among adolescents before (September 2019) and during the COVID-19 pandemic (June 2020) in Hong Kong (25). In a representative sample of Chines college students, survey during initial breakout of the COVID-19 pandemic and after decline in the second wave revealed that psychological distress significantly reduced after the peak of pandemic, but persisted in some students (49). These differences may be due to the study sample as well as the country in which the study was conducted, considering the different approaches of the governments as well as the impact of the pandemic on citizens.

Regarding alcohol consumption, the evidence is heterogeneous to date. An increase in total AUDIT-C scores during the pandemic compared to pre-COVID-19 was shown in a sample of vulnerable groups (people with self-reported mental health problems) (50). While an increased alcohol consumption during the pandemic, as found in our study, was reported by several other studies (51), a decreased or unchanged consumption was also observed (52, 53). The inconsistent results may be explained by the differences in study population as well as measurement time (beginning of the pandemic etc.) and different methods of assessment. Surprisingly, more direct contacts, higher self-efficacy, social support as well as resilience were positive predictors of higher scores for AUDIT-C, although no direct contacts as well as lower social support and self-efficacy predicted the higher levels of alcohol consumption in the first survey (10). Multiple social factors could account for these findings: During the first lockdown most of the people showed a high determination and motivation to follow the restrictions mandated by the government hoping that the pandemic will not last long. However, with the second wave many people noticed high exhaustion and hereby started not protecting themselves as carefully as in the first pandemic. Voluntary protective behavior was found to be less common especially among younger adults (< 30 years) (52). For this reason, it is possible that social gatherings took place more often, which students showing more resilience and having more social support and self-efficacy attended and consumed more alcohol there. Statistically significantly more participants reported consuming drugs in the second study. However, more participants in the second study indicated less drug consumption since the pandemic. Many studies highlight increased substance use since the onset of the pandemic (22, 54, 55).

This study also revealed an unchanged symptom severity of bulimia nervosa. In the existing literature there are inconsistent results regarding eating disorders: Breiner et al. demonstrated no significant changes in global scores of EDE-Q (Eating Disorder Examination Questionnaire) as well as self-induced vomiting and laxative use from prior to during the pandemic (56). On the other hand, some studies reported an increase in eating disorder symptoms (25, 57).

Because of higher mortality rates and several somatic comorbidities, the older adults were more in focus than the younger age groups at the beginning of the pandemic. However, findings showed that younger adults are experiencing more mental health symptoms, including depressive and anxiety symptoms, during the pandemic than older adults (58). Also, the present study highlights an increase in mental health symptoms among university students after ~one year of the pandemic. Overall, the existing evidence to date provides only a limited picture on changes in mental health of university students during the pandemic. Therefore, further studies investigating changes in mental health outcomes and associated factors are needed. Moreover, public health measures concerning the mental health of young adults should be promoted and adapted during crises like the pandemic. Through the expansion of online intervention possibilities barriers such as contacting medical professionals can be alleviated (59, 60).

Strengths of this study include the large sample size and the availability of a large comparison sample from 2020. Thus, changes in mental health outcomes during the course of the pandemic could be evaluated. The survey was conducted in a large German university including a wide range of faculties and with a high response rate, allowing for generalizability of the results to German students. Further, validated and well-established instruments were used.

This study has several limitations. First, change in depressive symptoms and symptoms of eating disorders as well as alcohol and drug consumption were assessed using cross-sectional student cohorts in 2020 and 2021, respectively. Because of the anonymity condition, it was not possible to identify the participants who participated in the online survey in 2020. Of note is that only 34.8% (*n* = 1,965) of the final sample stated participating in the first survey from 2020. Second, the results are limited to a single German university, therefore generalizability may be limited, also given the fact that solely self-report measures had been applied. Third, it is not possible to draw causal conclusions because of the observational nature of the study.

## Conclusion

The present study reveals an increase in severities of depressive symptoms, including suicidal ideation, as well as drug and alcohol consumption among students of University Leipzig. Being diverse and of younger age, higher perceived stress and loneliness were risk factors for higher depressive and eating disorder symptoms, respectively, as well as alcohol consumption. Universities and health care policy should recognize and address mental health issues of young adults especially during times of crisis. Targeted preventive programs as well as easy-to-access measures to support students with mental health problems should be prepared and performed accordingly. Against this background, a return to pre-pandemic face-to-face seminars and course in universities, social activities and other events and “campus life”—covered by a hygiene concept and taking into account that a vaccination is broadly available nowadays, should be favored, whenever possible.

## Data Availability Statement

The raw data supporting the conclusions of this article will be made available by the authors, without undue reservation.

## Ethics Statement

The studies involving human participants were reviewed and approved by Ethics Statement. The Ethics Committee of the Medical Faculty, University of Leipzig, waived ethical approval for this study because of anonymity of the survey (08.03.2021). The participants provided informed consent to participate in this study. The patients/participants provided their written informed consent to participate in this study.

## Author Contributions

EK and ED-S equally contributed to the current paper and share first authorship. EK, ED-S, and CR-K conceptualized the study and constructed the questionnaires. ED-S and EK performed data analyses and drafted the initial version of the manuscript. SB and CR-K critically reviewed and revised the manuscript. All authors have read and approved the content of the final manuscript.

## Funding

We also acknowledge support from Leipzig University for Open Access Publishing.

## Conflict of Interest

The authors declare that the research was conducted in the absence of any commercial or financial relationships that could be construed as a potential conflict of interest.

## Publisher's Note

All claims expressed in this article are solely those of the authors and do not necessarily represent those of their affiliated organizations, or those of the publisher, the editors and the reviewers. Any product that may be evaluated in this article, or claim that may be made by its manufacturer, is not guaranteed or endorsed by the publisher.

## References

[1] Salari N Hosseinian-Far A Jalali R Vaisi-Raygani A Rasoulpoor S Mohammadi M. Prevalence of stress, anxiety, depression among the general population during the COVID-19 pandemic: a systematic review and meta-analysis. Global Health. (2020) 16:57. 10.1186/s12992-020-00589-w 32631403 PMC7338126

[2] Sanyaolu A Okorie C Marinkovic A Patidar R Younis K Desai P. Comorbidity and its impact on patients with COVID-19. SN Compr Clin Med. (2020) 2:1–8. 10.1007/s42399-020-00363-4 32838147 PMC7314621

[3] Kessler RC Bromet EJ. The epidemiology of depression across cultures. Annu Rev Public Health. (2013) 34:119–38. 10.1146/annurev-publhealth-031912-114409 23514317 PMC4100461

[4] Pedrelli P Nyer M Yeung A Zulauf C Wilens T. College students: mental health problems and treatment considerations. Acad Psychiatry. (2015) 39:503–11. 10.1007/s40596-014-0205-9 25142250 PMC4527955

[5] Luo W Zhong B-L Chiu HF-K. Prevalence of depressive symptoms among Chinese university students amid the COVID-19 pandemic: a systematic review and meta-analysis. Epidemiol Psychiatr Sci. (2021) 30:e31. 10.1017/S2045796021000202 33766163 PMC8047400

[6] Wathelet M Duhem S Vaiva G Baubet T Habran E Veerapa E. Factors associated with mental health disorders among university students in France confined during the COVID-19 pandemic. JAMA Netw Open. (2020) 3:e2025591. 10.1001/jamanetworkopen.2020.25591 33095252 PMC7584927

[7] Supke M Hahlweg PK Kelani K Muschalla PB Schulz PW. Mental Health, Partnerships, and Sexual Behavior of German Students After the Third Wave During the Corona Pandemic. (2021). [Preprint] 10.21203/rs.3.rs-842233/v1 36125795

[8] Kecojevic A Basch CH Sullivan M Davi NK. The impact of the COVID-19 epidemic on mental health of undergraduate students in New Jersey, cross-sectional study. PLoS ONE. (2020) 15:e0239696. 10.1371/journal.pone.0239696 32997683 PMC7526896

[9] Islam MA Barna SD Raihan H Khan MN Hossain MT. Depression and anxiety among university students during the COVID-19 pandemic in Bangladesh: a web-based cross-sectional survey. PLoS ONE. (2020) 15:e0238162. 10.1371/journal.pone.0238162 32845928 PMC7449469

[10] Kohls E Baldofski S Moeller R Klemm S-L Rummel-Kluge C. Mental health, social and emotional well-being, and perceived burdens of university students during COVID-19 pandemic lockdown in Germany. Front Psychiatry. (2021) 12:643957. 10.3389/fpsyt.2021.643957 33889102 PMC8055863

[11] WHO/Europe. Suicide a Leading Cause of Death Among Young Adults in High-Income Countries (2014). Availablee online at: https://www.euro.who.int/en/health-topics/noncommunicable-diseases/mental-health/news/news/2014/09/suicide-a-leading-cause-of-death-among-young-adults-in-high-income-countries (accessed July 26, 2021).

[12] Radeloff D Papsdorf R Uhlig K Vasilache A Putnam K von Klitzing K. Trends in suicide rates during the COVID-19 pandemic restrictions in a major German city. Epidemiol Psychiatr Sci. (2021) 30:e16. 10.1017/S2045796021000019 33461639 PMC7889841

[13] Pirkis J John A Shin S DelPozo-Banos M Arya V Analuisa-Aguilar P. Suicide trends in the early months of the COVID-19 pandemic: an interrupted time-series analysis of preliminary data from 21 countries. Lancet Psychiatr. (2021) 8:579–88. 10.1016/S2215-0366(21)00091-2 33862016 PMC9188435

[14] Leske S Kõlves K Crompton D Arensman E Leo Dde. Real-time suicide mortality data from police reports in Queensland, Australia, during the COVID-19 pandemic: an interrupted time-series analysis. Lancet Psychiatr. (2021) 8:58–63. 10.1016/S2215-0366(20)30435-1 33212023 PMC7836943

[15] O'Connor RC Wetherall K Cleare S McClelland H Melson AJ Niedzwiedz CL. Mental health and well-being during the COVID-19 pandemic: longitudinal analyses of adults in the UK COVID-19 mental health & wellbeing study. Br J Psychiatry. (2020):1–8. 10.31234/osf.io/r8cdw 33081860 PMC7684009

[16] Czeisler MÉ Lane RI Petrosky E Wiley JF Christensen A Njai R. Mental health, substance use, and suicidal ideation during the COVID-19 pandemic - United States, June 24-30, 2020. MMWR. (2020) 69:1049–57. 10.15585/mmwr.mm6932a1 32790653 PMC7440121

[17] Jackson C Sundaram V. ‘I have a sense that it's probably quite bad … but because I don't see it, I don't know’: staff perspectives on ‘lad culture’ in higher education. Gender Educ. (2021) 33:435–50. 10.1080/09540253.2018.1501006

[18] Lechner WV Laurene KR Patel S Anderson M Grega C Kenne DR. Changes in alcohol use as a function of psychological distress and social support following COVID-19 related university closings. Addict Behav. (2020) 110:106527. 10.1016/j.addbeh.2020.106527 32679435 PMC7319610

[19] Graupensperger S Jaffe AE Fleming CN Kilmer JR Lee CM Larimer ME. Changes in college student alcohol use during the COVID-19 pandemic: are perceived drinking norms still relevant? Emerg Adulthood. (2021) 1–10. 10.1177/2167696820986742 PMC8664006 34900403

[20] Bollen Z Pabst A Creupelandt C Fontesse S Lannoy S Pinon N. Prior drinking motives predict alcohol consumption during the COVID-19 lockdown: a cross-sectional online survey among Belgian college students. Addict Behav. (2021) 115:106772. 10.1016/j.addbeh.2020.106772 33418433

[21] Bertrand L Shaw KA Ko J Deprez D Chilibeck PD Zello GA. The impact of the coronavirus disease 2019 (COVID-19) pandemic on university students' dietary intake, physical activity, and sedentary behaviour. Appl Physiol Nutr Metab. (2021) 46:265–72. 10.1139/apnm-2020-0990 33449864

[22] Yehudai M Bender S Gritsenko V Konstantinov V Reznik A Isralowitz R. COVID-19 fear, mental health, and substance misuse conditions among university social work students in Israel and Russia. Int J Ment Health Addict. (2020) 1–8. 10.1007/s11469-020-00360-7 32837438 PMC7338139

[23] Busse H Buck C Stock C Zeeb H Pischke CR Fialho PM. Engagement in health risk behaviours before and during the COVID-19 pandemic in German University students: results of a cross-sectional study. Int J Environ Res Public Health. (2021) 18:1410. 10.3390/ijerph18041410 33546344 PMC7913592

[24] Prowse R Sherratt F Abizaid A Gabrys RL Hellemans KG Patterson ZR. Coping with the COVID-19 pandemic: examining gender differences in stress and mental health among university students. Front Psychiatry. (2021) 12:650759. 10.3389/fpsyt.2021.650759 33897499 PMC8058407

[25] Zhou Y Wade TD. The impact of COVID-19 on body-dissatisfied female university students. Int J Eat Disord. (2021) 54:1283–8. 10.1002/eat.23521 33851442 PMC8250175

[26] Flaudias V Iceta S Zerhouni O Rodgers RF Billieux J Llorca P-M. COVID-19 pandemic lockdown and problematic eating behaviors in a student population. J Behav Addict. (2020) 9:826–35. 10.1556/2006.2020.00053 32976112 PMC8943668

[27] Ligus K Fritzson E Hennessy EA Acabchuk RL Bellizzi K. Disruptions in the management and care of university students with preexisting mental health conditions during the COVID-19 pandemic. Transl Behav Med. (2021) 11:802–7. 10.1093/tbm/ibab020 33749756 PMC8083644

[28] Husky MM Kovess-Masfety V Gobin-Bourdet C Swendsen J. Prior depression predicts greater stress during Covid-19 mandatory lockdown among college students in France. Compr Psychiatry. (2021) 107:152234. 10.1016/j.comppsych.2021.152234 33706216

[29] Fruehwirth JC Biswas S Perreira KM. The Covid-19 pandemic and mental health of first-year college students: examining the effect of Covid-19 stressors using longitudinal data. PLoS ONE. (2021) 16:e0247999. 10.1371/journal.pone.0247999 33667243 PMC7935268

[30] Zimmermann M Bledsoe C Papa A. The Impact of the COVID-19 Pandemic on College Student Mental Health: A Longitudinal Examination of Risk and Protective Factors. (2020). 10.31234/osf.io/2y7hu 34763271 PMC8556872

[31] Volken T Zysset A Amendola S Klein Swormink A Huber M von Wyl A. Depressive symptoms in Swiss University students during the COVID-19 pandemic and its correlates. Int J Environ Res Public Health. (2021) 18:1458. 10.3390/ijerph18041458 33557193 PMC7913894

[32] Reznik A Gritsenko V Konstantinov V Yehudai M Bender S Shilina I. First and second wave COVID-19 fear impact: Israeli and Russian social work student fear, mental health and substance use. Int J Ment Health Addict. (2021) 1–8. 10.1007/s11469-020-00481-z 33551690 PMC7852475

[33] Meda N Pardini S Slongo I Bodini L Zordan MA Rigobello P. Students' mental health problems before, during, and after COVID-19 lockdown in Italy. J Psychiatr Res. (2021) 134:69–77. 10.1016/j.jpsychires.2020.12.045 33360865

[34] Robert Koch Institute. Coronavirus Disease 2019 (COVID-19) Weekly Situation Report of the Robert Koch Institute (2021). Availablee online at: https://www.rki.de/DE/Content/InfAZ/N/Neuartiges_Coronavirus/Situationsberichte/Apr_2021/2021-04-08-en.pdf?__blob=publicationFile (accessed July 26, 2021).

[35] Kroenke K Spitzer RL Williams JB. The PHQ-9: validity of a brief depression severity measure. J Gen Intern Med. (2001) 16:606–13. 10.1046/j.1525-1497.2001.016009606.x 11556941 PMC1495268

[36] Higgins-Biddle JC Babor TF. A review of the Alcohol Use Disorders Identification Test (AUDIT), AUDIT-C, and USAUDIT for screening in the United States: past issues and future directions. Am J Drug Alcohol Abuse. (2018) 44:578–86. 10.1080/00952990.2018.1456545 29723083 PMC6217805

[37] Bauer S Winn S Schmidt U Kordy H. Construction, scoring and validation of the Short Evaluation of Eating Disorders (SEED). Eur Eat Disorders Rev. (2005) 13:191–200. 10.1002/erv.63725855820

[38] Kendel F Spaderna H Sieverding M Dunkel A Lehmkuhl E Hetzer R. Eine deutsche Adaptation des ENRICHD Social Support Inventory (ESSI). Diagnostica. (2011) 57:99–106. 10.1026/0012-1924/a000030

[39] Hughes ME Waite LJ Hawkley LC Cacioppo JT. A short scale for measuring loneliness in large surveys: results from two population-based studies. Res Aging. (2004) 26:655–72. 10.1177/0164027504268574 18504506 PMC2394670

[40] Schwarzer R Jerusalem M. Skalen zur Erfassung von Lehrer- und Schülermerkmalen. Dokumentation der psychometrischen Verfahren im Rahmen der Wissenschaftlichen Begleitung des Modellversuchs Selbstwirksame Schulen: Testbeschreibung SWE (1999). p. 4. Available online at: http://www.fu-berlin.de/gesund/ (accessed February 27, 2021).

[41] Smith BW Dalen J Wiggins K Tooley E Christopher P Bernard J. The brief resilience scale: assessing the ability to bounce back. Int J Behav Med. (2008) 15:194–200. 10.1080/10705500802222972 18696313

[42] Cohen S Kamarck T Mermelstein R. A global measure of perceived stress. J Health Soc Behav. (1983) 24:385. 10.2307/2136404 6668417

[43] Cohen J. Statistical Power Analysis for the Behavioral Sciences. London: Routledge. (2013). 10.4324/9780203771587

[44] Feter N Caputo EL Doring IR Leite JS Cassuriaga J Reichert FF. Sharp increase in depression and anxiety among Brazilian adults during the COVID-19 pandemic: findings from the PAMPA cohort. Public Health. (2021) 190:101–7. 10.1016/j.puhe.2020.11.013 33387848 PMC7773543

[45] Ettman CK Abdalla SM Cohen GH Sampson L Vivier PM Galea S. Prevalence of depression symptoms in US adults before and during the COVID-19 pandemic. JAMA Netw Open. (2020) 3:e2019686. 10.1001/jamanetworkopen.2020.19686 32876685 PMC7489837

[46] Bendau A Kunas SL Wyka S Petzold MB Plag J Asselmann E. Longitudinal changes of anxiety and depressive symptoms during the COVID-19 pandemic in Germany: the role of pre-existing anxiety, depressive, and other mental disorders. J Anxiety Disord. (2021) 79:102377. 10.1016/j.janxdis.2021.102377 33662702 PMC9758512

[47] Xu Y Su S Jiang Z Guo S Lu Q Liu L. Prevalence and risk factors of mental health symptoms and suicidal behavior among university students in Wuhan, China during the COVID-19 pandemic. Front Psychiatry. (2021) 12:695017. 10.3389/fpsyt.2021.695017 34326787 PMC8313758

[48] Efstathiou V Michopoulos I Yotsidi V Smyrnis N Zompola C Papadopoulou A. Does suicidal ideation increase during the second COVID-19 lockdown? Psychiatry Res. (2021) 301:113990. 10.1016/j.psychres.2021.113990 34020218 PMC9225821

[49] Gong S Li LZ Wang S. Youth mental health before and after the control of the coronavirus disease 2019: a nationally representative cohort study of Chinese college students. J Affect Disord Rep. (2021) 3:100066. 10.1016/j.jadr.2020.100066 PMC8995102 35434689

[50] Naughton F Ward E Khondoker M Belderson P Marie Minihane A Dainty J. Health behaviour change during the UK COVID-19 lockdown: Findings from the first wave of the C-19 health behaviour and well-being daily tracker study. Br J Health Psychol. (2021) 26:624–43. 10.1111/bjhp.12500 33410229 PMC9291054

[51] Pollard MS Tucker JS Green HD. Changes in adult alcohol use and consequences during the COVID-19 pandemic in the US. JAMA Netw Open. (2020) 3:e2022942. 10.1001/jamanetworkopen.2020.22942 32990735 PMC7525354

[52] Wang Y Lu H Hu M Wu S Chen J Wang L. Alcohol Consumption in China before and during COVID-19: preliminary results from an online retrospective survey. Front Psychiatry. (2020) 11:597826. 10.3389/fpsyt.2020.597826 33324263 PMC7723925

[53] Kekäläinen T Hietavala E-M Hakamäki M Sipilä S Laakkonen EK Kokko K. Personality traits and changes in health behaviors and depressive symptoms during the COVID-19 pandemic: a longitudinal analysis from pre-pandemic to onset and end of the initial emergency conditions in Finland. Int J Environ Res Public Health. (2021) 18:7732. 10.3390/ijerph18157732 34360025 PMC8345535

[54] Betsch C Korn L Felgendreff L Eitze S Schmid P Sprengholz P. German COVID19 Snapshot Monitoring (COSMO) – Welle 34. (2021). Available online at: https://projekte.uni-erfurt.de/cosmo2020/web/summary/34/ (accessed August 05, 2021).

[55] Horigian VE Schmidt RD Feaster DJ. Loneliness, mental health, and substance use among US young adults during COVID-19. J Psychoactive Drugs. (2021) 53:1–9. 10.1080/02791072.2020.1836435 33111650

[56] Breiner CE Miller ML Hormes JM. Changes in eating and exercise behaviors during the COVID-19 pandemic in a community sample: a retrospective report. Eat Behav. (2021) 42:101539. 10.1016/j.eatbeh.2021.101539 34245981 PMC9760093

[57] Kim H Rackoff GN Fitzsimmons-Craft EE Shin KE Zainal NH Schwob JT. College mental health before and during the COVID-19 pandemic: results from a nationwide survey. Cogn Ther Res. (2021):1–10. 10.1007/s10608-021-10241-5 34177004 PMC8214371

[58] Gambin M Sekowski M Wozniak-Prus M Wnuk A Oleksy T Cudo A. Generalized anxiety and depressive symptoms in various age groups during the COVID-19 lockdown in Poland. Specific predictors and differences in symptoms severity. Compr Psychiatry. (2021) 105:152222. 10.1016/j.comppsych.2020.152222 33388494

[59] Scholl J Kohls E Görges F Steinbrecher M Baldofski S Moessner M. Acceptability and feasibility of the transfer of face-to-face group therapy to online group chats in a psychiatric outpatient setting during the COVID-19 pandemic: longitudinal observational study. JMIR Form Res. (2021) 5:e27865. 10.2196/27865 34161252 PMC8315157

[60] Baldofski S Kohls E Bauer S Becker K Bilic S Eschenbeck H. Efficacy and cost-effectiveness of two online interventions for children and adolescents at risk for depression (Emotion trial): study protocol for a randomized controlled trial within the ProHEAD consortium. Trials. (2019) 20:53. 10.1186/s13063-018-3156-8 30646944 PMC6334409

